# Comparative Evaluation of Occlusion before and after Soft Tissue Mobilization in Patients with Temporomandibular Disorder—Myofascial Pain with Referral

**DOI:** 10.3390/ijerph18126568

**Published:** 2021-06-18

**Authors:** Joanna Kuć, Krzysztof Dariusz Szarejko, Maria Gołębiewska

**Affiliations:** 1 Department of Prosthodontics, Medical University of Bialystok, 24A M. Sklodowskiej-Curie Street, 15-274 Bialystok, Poland; 2 Private Health Care, Physical Therapy and Rehabilitation, Bialystok, 79 Warsaw Street, 15-201 Bialystok, Poland; biuro@rehabilitacja-lecznicza.pl; 3 Department of Dental Techniques, Medical University of Bialystok, 13 Washington Street, 15-269 Bialystok, Poland; maria.golebiewska@umb.edu.pl

**Keywords:** biotensegrity, myofascial pain with referral, occlusion, soft tissue mobilization, temporomandibular joint, T-scan III

## Abstract

The aim of the study was to evaluate occlusal parameters in patients with myofascial pain with referral before and after soft tissue mobilization. The study group consisted of 50 people (37 females and 13 males, average age 23.36 ± 2.14 years) diagnosed with myofascial pain with referral. All patients underwent triplicate soft tissue mobilization. Occlusal parameters were evaluated six times, before and after each treatment, using T-scan III. A decreasing tendency of the occlusion time was observed after the first, second, and third therapy. After the third treatment, the mean occlusion time in the entire study group was 0.119 s. The 1st soft tissue mobilization shortened both right and left disclusion times to 0.181 s and 0.185 s, respectively. After the third treatment, these parameters amounted to 0.159 s and 0.165 s, respectively. The Friedman test for the entire study group indicated that soft tissue mobilization altered the occlusion time and both disclusion times (*p* < 0.05). In conclusion, soft tissue mobilization affects biotensegrity of the masticatory system, thus modifying occlusal parameters. The occlusion time and both disclusion times cannot be considered as cofactors of the existing temporomandibular disorders—myofascial pain with referral.

## 1. Introduction

The masticatory system is defined as a functional complex characterized by a multitude of components including bones, teeth, soft tissues, muscles, tendons, ligaments, and discs [1]. Two temporomandibular joints enable the motion of the mandible within a range of six degrees of movements (translation along and rotation around three mutually perpendicular axes) [1,2]. The motion of the mandible triggers the coactivation of 16 groups of mandibular muscles, which results in cumulative force interplay within the teeth [2]. Hypothetically, there are unlimited patterns of muscle coactivation to provide a desired occlusal load or jaw movement [2]. In fact, repeated occlusal contacts and jaw movements remain in accordance with regular motor command paths created by the brain stem during function (central pattern generator) [2,3].

Dental occlusion reflects unique information contained in a center in the brain, specialized in summation and integration of neurological signaling originating from periodontal, dental, and soft tissue receptors. This complex is permanently controlled by the central nervous system (CNS) to adjust and improve mandible position and motion in accordance with peripheral inputs [4,5]. Within this system, there is sensorimotor neuroplasticity, which largely determines individual adaptation to occlusal and oral changes resulting from dental treatments [5].

The relationship between dental occlusion and temporomandibular disorders (TMDs) still remains controversial, inconclusive, and not fully examined [5,6,7]. Currently, etiological factors are based mainly on behavioral, psychological, and neurological components [6]. Diagnostic Criteria for Temporomandibular Disorders (DC/TMD) provide an appropriate, valid, and reliable clinical tool to perform differential diagnosis with respect to physical and biopsychosocial condition (Axes I and II, respectively) [5,8,9,10,11]. This protocol creates a modern approach to the etiology of TMDs. Treatment modalities for TMDs include pharmacotherapy (e.g., analgesics, non-steroidal anti-inflammatory drugs, anxiolytics, anti-depressants, Imotun, CBD), occlusal appliances, counseling, physical therapy, manual therapy, therapeutic exercises, arthroscopy, arthrocentesis, joint injections of hyaluronic acid, and muscle injections of botulinum toxin [12,13,14,15,16,17,18].

The latest physiological concept considers the temporomandibular complex from the perspective of biotensegrity. According to this theory, the mandible is suspended within a tensioned network, which spreads out anatomically much further than usually assumed [19]. Furthermore, in this biological interface, it is anatomical structures that are primarily responsible for motion control. This evolutionarily conditioned system enables a quick response to functional changes and provides a more coherent model of joint physiology [19]. Biotensegrity interplays with elastic deformation of the bone. Under functional loading, the human mandible demonstrates flexible biomechanical behavior, which results in the flexion at the site of symphysis, entailing the reduction of the distance between left and right mandibular ramus and thus leads to a close-up of the lateral segments of the lower dental arch [20]. It is also well known that dynamic loading implicates the bone metabolic activity. During speech, masticatory muscles trigger stress effect throughout the mandible [21]. It is assumed that loading frequency while speaking may be from three to five times greater than that of mastication [21]. Considering anatomical relationships and biomechanical condition of the mandible, the highest strains arising from speech activity probably occurs within the chin [21]. This suggests that speech creates a stress pattern that triggers bone modeling, mainly in the anterior part of the mandible [21].

Biological systems based on biotensegrity are characterized by a high level of resiliency to external disturbances. Through appropriate changes in tension/compression distribution, they maintain a balance between self-stabilization and immediate ability to respond to the load affecting them during every motion. Biotensegrity could be defined as a global balance between compressional and tensional forces—“the balance of unseen forces” [19]. It represents internal stability of any system of forces embedded in nature [22].

In the light of the above, it may be concluded that the condition of the temporomandibular joint as well as the entire masticatory system is presumably modulated by the variability of tension/compression distribution. Each part of the masticatory system contributes to its kinematic behavior with superior control from the nervous system. The main clinical point is that, according to the theory of biotensegrity, compressional forces are not directly transferred through the condyle, disc, and glenoid fossa. This approach remains in contrast to the classical theory of the third-class levers supported by the force-vector dependence [23]. The standard mechanical theory follows typical linear stress-strain curves. For tensegrity and living tissues, the non-linear dynamics is assigned [24].

These two perspectives may mirror controversies in recognizing dental occlusion as a potential risk factor in the development of TMDs and reporting research discrepancies. It is possible that changes within anatomical structures (disc perforation, erosion, degeneration) appear when a certain level of resiliency to external disturbances in biotensegrity-related systems is exceeded (cut-off point in the non-linear curve). This could suggest a direct link between occlusion and TMDs. Disturbances of self-regulation result in decompensation, i.e., loss of homeostasis. In conclusion, biotensegrity provides balance—enormous potential adaptation and compensation in one—and research findings depend on the advancement level of the captured changes.

Considering that soft tissue mobilization is a kind of external load, which leads to the release of tension and stress of myofascial components, achievement of new balance between tensional and compressional forces and changes in myofascial force distribution in a non-invasive manner, and remembering that soft tissue mobilization is closely linked to biotensegrity, it was hypothesized that there is at least one significant difference between pre- and post-treatment values of occlusal parameters in patients with temporomandibular disorder—myofascial pain with referral. As a dysfunction of multifactorial nature, myofascial pain with referral is always related with trigger points of the head and neck. This condition reflects a combination of sensory experiences, motor reactions, and autonomic symptoms, including local and referred pain [25,26,27].

The primary aim of the study was to evaluate occlusal parameters as cofactors of the existing TMDs in patients with myofascial pain with referral.

The second objective was to assess the influence of soft tissue mobilization on occlusion time, disclusion time, and occlusal loads.

## 2. Materials and Methods

### 2.1. Ethical Issues

The approval for the study was obtained from the Bioethics Committee of the Medical University of Bialystok, Poland (permit number: R-I-002/322/2016). The research was conducted in compliance with the Declaration of Helsinki of the World Medical Association and Guidelines for Good Clinical Practice. All the patients participated in the study voluntarily and were provided with comprehensive information about the nature, scope of activities, and course of clinical procedures. Every participant was enrolled in the study upon a prior written consent and had the right to withdraw from the experiment at any stage of the research, without consequences.

### 2.2. Subjects and the Size of the Sample

The study group consisted of 50 generally healthy people (37 women and 13 men) with complete natural dentition. The average age of the participants was 23.36 ± 2.14 years. The subjects had been referred to the Department of Prosthodontics at the Medical University of Bialystok, Poland. All of them underwent a clinical examination according to the Diagnostic Criteria for Temporomandibular Disorders (DC/TMD) and were diagnosed with myofascial pain with referral [9].

#### 2.2.1. Inclusion Criteria

- At least eight points of craniofacial and/or craniomandibular pain (according to the Visual Analog Scale, VAS),
- Full natural dentition, including class I of Angle’s Molar Classification and canine position,
- No history of orthodontic treatment or retention status within three years after the completion of the treatment.

#### 2.2.2. Exclusion Criteria

- History of craniofacial and/or craniomandibular injuries,
- Any special surgical treatment within the craniofacial and/or craniomandibular region in the past,
- History of occlusal splint therapy,
- Any previous prosthetic treatment,
- Any previous physiotherapy within the craniofacial and/or craniomandibular region,
- Health concerns which could affect the functioning of the masticatory muscles,
- Metabolic diseases,
- Medications (long-lasting intake in the past and at present).

The study group was described in detail in previous publications [25,26,27].

### 2.3. General Description of the Method

All patients underwent a thorough clinical examination including:

- Functional examination of the masticatory system with respect to the Diagnostic Criteria for Temporomandibular Disorders (DC/TMD)—axes I and II [9]
- Functional analysis of dental occlusion (T-scan III system; Tekscan, Inc., Boston, MA, USA),
- Soft tissue mobilization [25],
- Statistical analysis using the Statistica 13.1 software (Statsoft Inc., Cracow, Poland), GraphPad Prism 8 (GraphPad Software, La Jolla, CA, USA) and PQStat Software v. 1.6.8. (PQStat Software, Poznań, Poland).

### 2.4. Functional Analysis of Dental Occlusion

#### 2.4.1. General Description of the T-Scan III System

**Figure 1:**
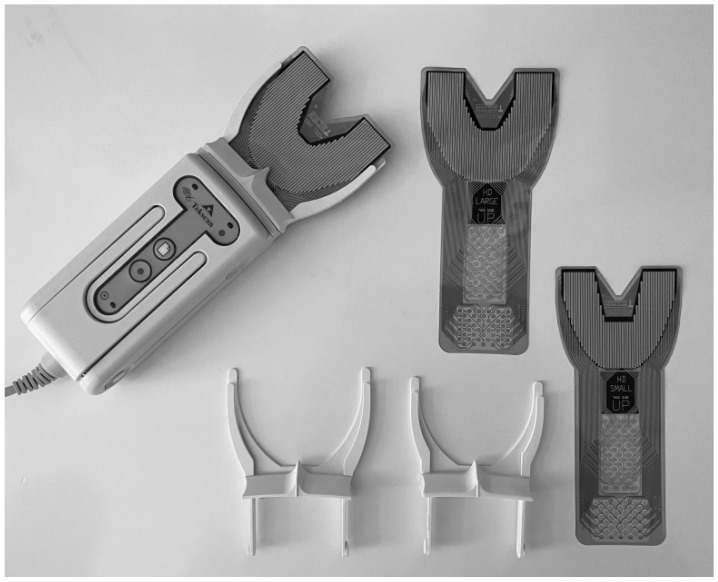
T-scan III panel (handle) that enables registration and a para-occlusal holder equipped with the sensor—100-micron-thick articulating foil.

Functional analysis of dental occlusion was performed using the T-scan III system (Tekscan, Inc., Boston, MA, USA), which consists of a special panel (handle) that enables registration and a para-occlusal holder equipped with a sensor in a form of 100-micron-thick articulating foil (Figure 1). The resiliency of the sensor to variations of occlusal forces enables the evaluation of the distribution of contact points in relation to the analyzed phase of the occlusion. Due to the width of the dental arches, the sensor is available in two sizes: small (S) and large (L).

The T-scan III system enables the evaluation of dynamic occlusion by registering functional parameters such as the occlusion time (OT), both right and left disclusion times (TDR and TDL, respectively) and balance, i.e., occlusal force distribution on the right and left side of clenched dental arches. The occlusion time is defined as the time from the first tooth contact to the maximum intercuspation of the upper and lower dental arches (the norm for natural dentition is <0.2 s). The right disclusion time (TDR) is the time from the maximum intercuspation of both dental arches to their complete lack of contact during the right laterotrusion (the norm for natural dentition is <0.4 s). The left disclusion time (TDL) is the time from the maximum intercuspation of the dental arches to their complete lack of contact during the left laterotrusion (the norm for natural dentition is <0.4 s) [28,29,30].

#### 2.4.2. T-Scan III Registration Procedure

T-scan III registration was conducted six times, always before and after the first, second, and third soft tissue mobilization. The subjects remained upright and sitting in the initial default position of the dental chair. The patients were asked to clench both dental arches together on the T-scan III sensor, hold this position for about 2 s, then open the mouth wide and repeat this procedure 3 times at a faster pace at 1-s intervals. Separate registrations were performed for lateral movements. After obtaining the maximum intercuspation and remaining in this position for about 2 s, the subjects made a movement of the mandible towards laterotrusion in accordance with the assumptions of canine guidance. There were 1 min breaks between consecutive registrations. A stopwatch was used for timing.

T-scan III registrations were conducted in the morning, in room temperature, under the conditions of suggestive relaxation, i.e., without background noises, side conversations or third parties accompanying the patient. Moreover, there were no visual, auditory and multisensory distractors (monitor light, the radio, smartphones, respectively). The entire procedure was conducted by the same trained examiner (J.K.).

### 2.5. Soft Tissue Mobilization

All the patients had soft tissue mobilization performed for 30 min. The treatments were implemented three times at weekly intervals under the same conditions. Trigger point therapy in the masseter and temporal muscles was applied along with the myofascial relaxation technique. Clinical procedures were performed by a qualified physiotherapist specializing in the field of general physiotherapy (the author, K. D. Sz.). The patient was laid on the table for a manual therapy and the specialist stood over the head of the treated person. The physiotherapist released the patient in silence, in a quiet room with no potential distracting factors.

The pressure was released from trigger points in the masseter and temporalis [31]. The trigger points were determined in the superficial layer of the masseters using the pincer method. One finger of the therapist remained inside the oral cavity within the cheek, and the other was held outside. Next, muscle palpation perpendicular to the direction of muscle fibers was performed in order to detect taut bands. External flat palpation was applied to the deep layer of the masseter. The same technique was implemented on temporal muscles.

**Figure 2:**
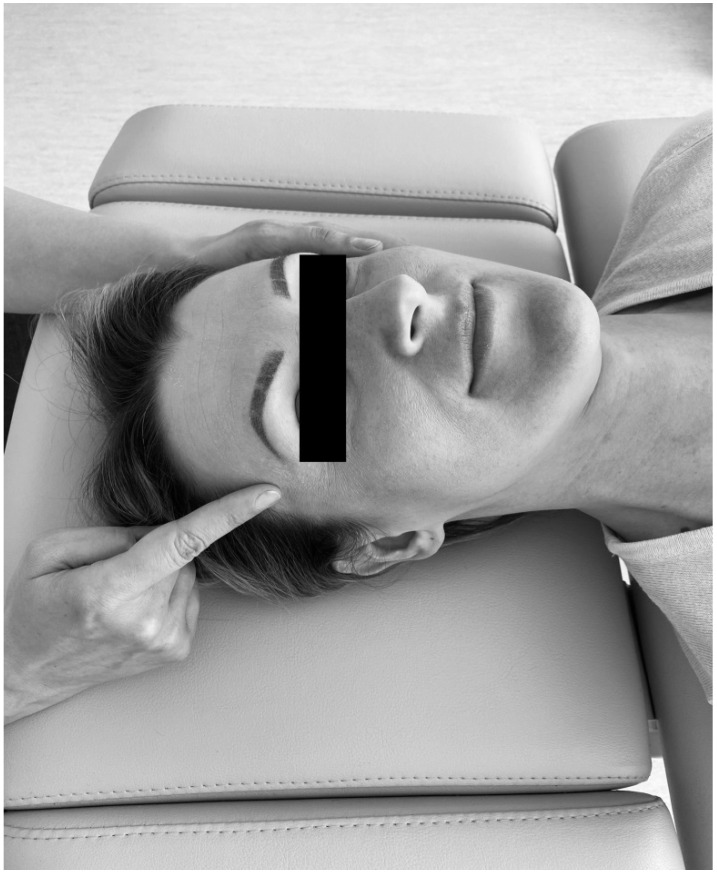
Unilateral temporal muscle mobilization.

**Figure 3:**
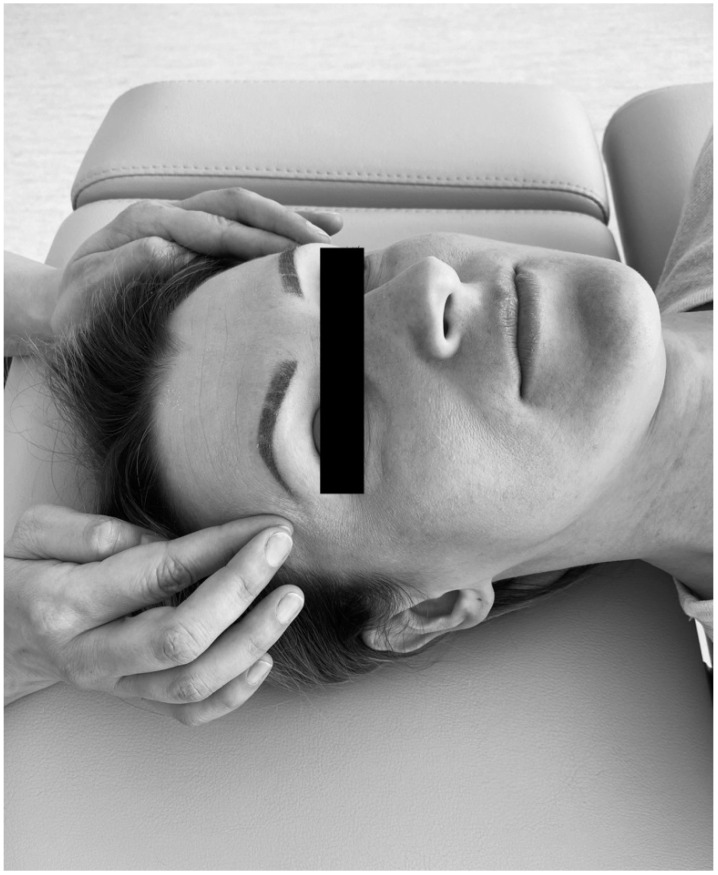
Bilateral temporal muscle mobilization.

The trigger point therapy consisted in initiating a slow increase of pressure on an active trigger point until reaching a tissue barrier [31]. The physiotherapist used one finger to contact the trigger point and an entire hand for contralateral head stabilization (Figure 2). For bilateral active trigger points, simultaneous treatment was provided with both hands (Figure 3). Soft tissue mobilization was started on the anterior edge of the muscle and proceeded towards its posterior margin. The therapy focused on the muscle areas that required to be released. The physiotherapist lengthened the muscle as far as the patient’s comfort allowed. Next, the specialist applied gentle, gradually increasing pressure on the trigger point until the finger reached a significant increase in tissue resistance (the barrier was engaged). It had been settled with patients that they might experience some discomfort, but pain was not acceptable [31]. This pressure was maintained (but not intensified) until the therapist felt the release of the tissue under the touching finger. Then the pressure was increased again. The extent of soft tissue laxity was adjusted accordingly to obtain a new barrier (the finger following the releasing tissue). Next, slight pressure was employed again to release more muscle tension under the touching finger. At that stage, it was considered acceptable to change the direction of pressure to achieve a better effect [31]. The technique of releasing pressure of trigger points involves adjusting to particular muscles of every individual patients and can be repeated for any taut muscle band [31]. The procedure was performed repeatedly until sensitivity and/or tension of the muscles decreased, or for 1.5 min, whichever occurred first.

**Figure 4:**
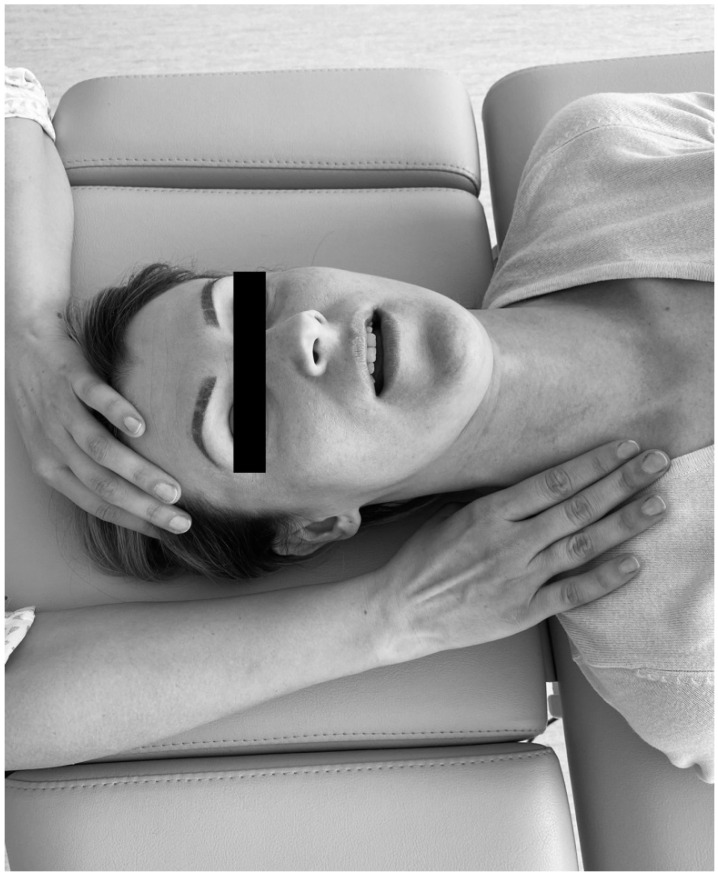
Upward–downward combined technique for the temporal muscle and the masseter [31].

**Figure 5:**
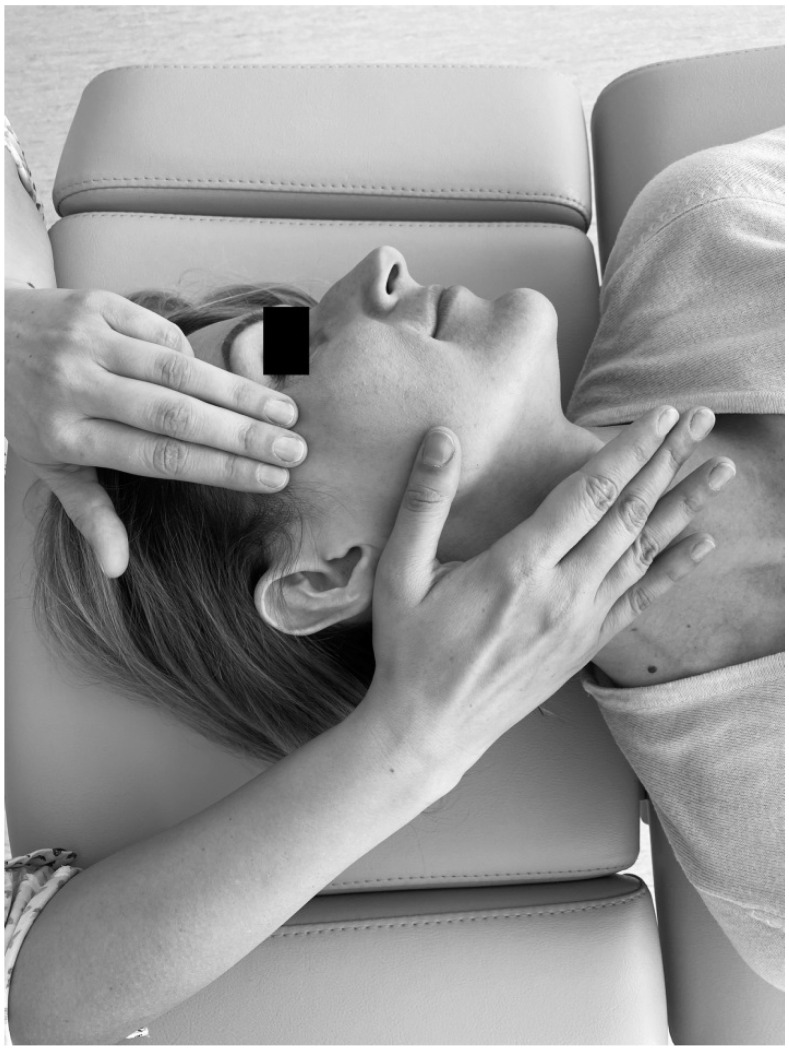
Masseter-oriented technique of soft tissue mobilization [31].

Myofascial relaxation was aimed at functionally releasing the fascia. The therapist placed one hand on the temporalis and took up the slack by upward traction (Figure 4) [31], simultaneously completing the myofascial release with the other hand via slow downward traction. Soft tissues were released from the temporal muscle and the masseter towards the platysma. In the other technique, the masseter muscle origin was stabilized in the zygomatic arch with one hand (Figure 5) [31]. The specialist moved the other hand along the muscle fibers—from the zygomatic arch to the mandibular margin. A slight pressure was simultaneously exerted to the posterior part of the mandible to detect laxity within the masseter. During this activity, the patient was asked to open the mouth wide and breathe deeply in order to boost the release of the muscles [31]. An initial balance was achieved after 1.5–2 min of impact. Then the physiotherapist treated the next tissue resistance point. The procedure was repeated several times (in approximately 2–5 cycles) until all the tension was released.

### 2.6. Statistical Analysis

Statistical analysis was performed using Statistica 13.1 (Statsoft, Inc., Cracow, Poland), GraphPad Prism 8 (GraphPad Software, La Jolla, CA, USA) and PQStat Software v. 1.6.8. (PQStat Software, Poznań, Poland). All the studied parameters (occlusion time, both right and left disclusion times, occlusal load) were evaluated in 6 periods—directly before and immediately after all three soft tissue mobilizations. The data was expressed as arithmetic mean, median and standard deviation. The Shapiro–Wilk test was applied to check the distribution of the obtained results. Parametric and non-parametric methods were implemented to determine whether soft tissue mobilization has an effect on the occlusion time, both right and left disclusion times and occlusal load. A one-way repeated measures ANOVA was applied in the case of normal distribution. Otherwise, the Friedman test was performed and Friedman statistic (F_r_), degrees of freedom (df), *p*-value and Kendall’s coefficient of concordance (W) were presented. Statistical significance was established at *p* < 0.05. Kendall’s W at a level of 0.1 indicated a small effect size. Moderate effect was established for W = 0.3 and strong effect size for W value above 0.5. Following the Friedman test, the Dunn–Bonferroni post hoc test was performed to compare all the treatments. In the case of the one-way repeated measures ANOVA, post hoc Tukey’s multiple comparison test was conducted. The Mann–Whitney U test was used to compare quantitative values between female and male patients at each stage of treatment.

## 3. Results

Before the first treatment, the average occlusion time (OT) in the entire study group (*n* = 50) was 0.191 s (Table 1). The values of the right and left disclusion times (TDR, TDL) were comparable and amounted to 0.209 s and 0.214 s, respectively. After the first mobilization, the OT dropped to 0.151 s. A similar decreasing tendency was observed after the second and third therapy (Table 1). The first soft tissue mobilization shortened TDR and TDL to 0.181 s and 0.185 s, respectively. After the third treatment, the mean occlusion time in the entire study group was 0.119 s. In the case of TDR and TDL, the values were similar and amounted to 0.159 s and 0.165 s, respectively (Table 1).

The Friedman test for the entire study group indicated that soft tissue mobilization altered the occlusion time (OT) and both disclusion times (TDR, TDL) (*p* < 0.05) (Table 1). The value of Kendall’s W coefficient was at least 0.12. For the occlusion time, this parameter oscillated around 0.28.

The Dunn–Bonferroni test revealed that in the occlusion time, there were statistically significant differences before and after the first treatment, before the first and after the second therapy, as well as before the first and after the third soft tissue mobilization (*p* < 0.05 adjusted to the Bonferroni correction) (Table 1). In the case of the right disclusion time, statistically significant differences were observed before the first and after the second mobilization as well as before the first and after the third soft tissue mobilization (*p* < 0.05 adjusted to the Bonferroni correction). The left disclusion time differed only in comparison with the first and third treatment (Table 1). 

In the group of women (*n* = 37), the average occlusion time was 0.182 s before the first treatment (Table 2). The values of the right and left disclusion times (TDR and TDL, respectively) were similar and amounted to 0.200 s and 0.206 s, respectively. After the first soft tissue mobilization, the OT was reduced to 0.152 s. An analogous decreasing tendency was observed after the second and third therapy (Table 2). The first soft tissue mobilization reduced TDR and TDL to 0.186 s and 0.187 s, respectively. After the third treatment, the mean occlusion time in the entire study group was 0.115 s. In the case of TDR and TDL, the values amounted to 0.152 s and 0.161 s, respectively (Table 2).

The Friedman test revealed statistically significant differences among the mean ranks of the occlusion time (OT) and both disclusion times (TDR, TDL) (*p* < 0.05) (Table 2).

The Dunn–Bonferroni test revealed that in the occlusion time, there were statistically significant differences before and after the first treatment, before the first and after the second therapy, as well as before the first and after the third soft tissue mobilization (*p* < 0.05 adjusted to the Bonferroni correction) (Table 2). In the case of the right disclusion time, statistically significant differences were reported before the first and after the second mobilization as well as before the first and after the third soft tissue mobilization (*p* < 0.05 adjusted to the Bonferroni correction). With respect to the left disclusion time, there were no statistically significant differences observed before the first and after the first, second and third soft tissue mobilizations, respectively (*p* > 0.05) (Table 2).

Before the first treatment, in the group of men (*n* = 13), the average occlusion time was 0.216 s (Table 3). The right and left disclusion times (TDR, TDL) were comparable and amounted to 0.236 s and 0.239 s, respectively. After the first soft tissue mobilization, the OT decreased to 0.145 s. A similar decreasing tendency was observed after the second and third therapy (Table 3). The first treatment reduced TDR and TDL to 0.165 s and 0.182 s, respectively. After the third soft tissue mobilization, the mean occlusion time in the male group was 0.129 s. In the case of TDR and TDL, the values amounted to 0.180 s and 0.177 s, respectively (Table 3).

The Friedman test revealed statistically significant differences among the mean ranks of the occlusion time (OT) and left disclusion time (TDL) (*p* < 0.05) (Table 3).

The Dunn–Bonferroni test demonstrated that only in the occlusion time, there were statistically significant differences before and after the first treatment, before the first and after the second therapy, as well as before the first and after the third soft tissue mobilization (*p* < 0.05 adjusted to the Bonferroni correction) (Table 3). With respect to the right and left disclusion times, there were no statistically significant differences before the first and after the first, second, and third soft tissue mobilizations, respectively (*p* > 0.05) (Table 3).

There were no statistically significant differences between females and males in terms of TO, TDR, and TDL at every stage of the research (*p* > 0.05) (Table 4).

Before the first soft tissue mobilization, the average occlusal load on the left side of both dental arches in the entire study group was 52.8%, and on the right side it was 47.2%. After the first, second, and third therapy, the distribution of the load was comparable to the initially registered values (Table 5). There was no statistically significant influence of soft tissue mobilization on the occlusal load distribution (*p* > 0.05) (Table 5). Similar trends were observed in the case of both the group of women and men (Table 6 and Table 7).

There were no statistically significant differences between females and males in terms of occlusal load distribution (%) in maximal intercuspation at every stage of the research (*p* > 0.05) (Table 8).

## 4. Discussion

Supporting systems in animal and vegetal bodies tend to receive and create forces (with external or internal origin) which cancel each other out, with the result equal to zero. It allows to restore the original anatomical structure [22]. This phenomenon reflects excellent force distribution based on the theory of biotensegrity [22]. Within this concept, the possibility of returning to original shape after being subject to force loading is determined by structural and functional balance. Changes introduced to the structure under the influence of forces result in a new design, new balance of forces, and thus new tensegrity [22].

Dental tensegrity can be evaluated from numerous perspectives—of one tooth, certain groups of teeth, a dental arch, or across the face [22]. The tensegrity of the dental arch is provided by interproximal surfaces, proper occlusion, biological forces including the occlusal load, activity of the cheeks, lips and other soft tissues, adaptive and functional bone remodeling, and age-related growth vectors originating from functional and esthetic adaptations [22].

Soft tissue mobilization offers a kind of an external load, which can lead to transient disturbances of myofascial components and tensegrity in one. As a consequence of myofascial release, changes in dental force distribution as well as improvement in movements of the mandible can be expected. This type of physiotherapeutic treatment may enable specialists to reach new balance between compressional and tensional forces.

In the presented study, soft tissue mobilization contributed to the reduction of the occlusion time (TO) as well as the right and left disclusion times (TDR, TDL) (Table 1, Table 2 and Table 3). Both before and after the treatment, occlusal parameters oscillated within the reference values (OT < 0.2 s, TDR < 0.4 s, TDL < 0.4 s) (Table 1, Table 2 and Table 3).

Changes of the occlusal parameters, induced by soft tissues mobilization, are probably triggered by relieving the tension and stress of the associated masticatory muscles and fascia [32]. As a consequence, the freedom and quality of motion as well as the range of eccentric movements of the mandible may improve. Moreover, the aspect of neuromuscular facilitation attributed to the repeated registrations of T-scan III cannot be excluded. Coordinated, regular movements of the mandible could influence the resetting and restoration of compressional and tensional forces within the myofascial system with all occlusal consequences. This phenomenon appears to be analogous to deprogramming and creating new muscle engrams [33,34]. T-scan III might prove useful in clinical procedures as an occlusal biofeedback [35,36]. This may be of great importance in the case of people who, due to occlusal reasons, have consciously changed their chewing pattern (one-sided chewing, bypassing selected anatomical units in the chewing cycle) and, consequently, overload the structures of the masticatory system. In some cases, occlusal equilibration may be necessary [37,38,39].

Peck et al. highlighted that in patients with acute inflammation and pain of the temporomandibular joint, attention should be paid to reducing jaw function rather than changing the occlusal scheme [2]. Furthermore, these authors stressed that the compression of the temporomandibular joint is the norm, which will not be altered by modification of the occlusal scheme [2]. Such an approach might stem from a hidden nature of biotensegrity. The above-mentioned soft tissue mobilization and occlusal biofeedback may be implemented as an alternative in some TMDs and pain management, leading to the establishment of new compressional/tensional force distribution and muscle engrams. This could possibly alleviate the patients’ symptoms and signs.

Other considerations indicate that remodeling capacity within the masticatory system facilitates adaptation to most occlusal functions and dysfunctions [6]. It seems to mirror the non-linear dynamics of a stress/strain curve, which is dedicated to tensegrity and living tissues [24]. Currently, there is insufficient evidence that occlusal adjustments may help to prevent or control TMDs. It is possible that there is no justification for occlusal corrections for the management or prevention of TMD [40]. This suggestion could be confirmed by the results of our research, in which, despite the existing TMDs–myofascial pain with referral, the obtained results for occlusal parameters remained at the level of reference values. It should be emphasized that the occlusion time is strongly associated with maximum intercuspation defined as the highest number of occlusal contacts. This position provides the most suitable occlusal load transfer, i.e., through the long axis of the teeth [41]. During the clenching, maximal intercuspation enables the most favorable dissipation of occlusal forces [41]. It is in line with the theory of biotensegrity [22] and imitates load dispersion similar to the Newton’s cradle steel balance. In the presented study, soft tissue mobilization seems to have optimized the activation timing of the muscles responsible for mandibular movements, thereby affecting the occlusion time in closure and disclusion time in lateral excursions. It is probable that the activation timing of individual muscle groups directly reflects the balance between compressional and tensional forces, indicating possible shifts in biotensegrity.

Sierpińska et al. reported that in patients with advanced tooth wear, prosthetic rehabilitation—including multipoint contacts and increased cuspal morphology—resulted in stability of the occlusal conditions [41]. The high degree of force equality per arch half seems to play a crucial role. These authors indicated that changes of the vertical occlusal dimension lead to the improvement of the occlusion time and a lack of significant changes in the case of disclusion time [41]. Furthermore, it has been evidenced that the activity of the masseter muscles is strongly positively associated with occlusal point contacts as compared to the flattener surface [41,42].

The lack of statistically significant differences in some TDR and TDL after first, second, or third mobilizations at certain stages of the study may indicate the lateralization of both occlusal and muscular disorders, clearly suggesting the direction of morphologically or/and functionally anchored dysfunction (*p* > 0.05) (Table 1, Table 2 and Table 3). Additional evaluation of the activity of the masticatory muscles responsible for lateral movements of the mandible might be necessary. This lateralization-related asymmetry passively distributes tension through muscle-fascial chains in the entire body. It could affect the human posture and reflect a general lack of equilibrium in individuals [43].

The absence of statistically significant differences with respect to gender confirmed the homogeneity of the study group, as well as the coherent nature of neuromuscular dependence determined by occlusal conditions (Table 4). The above-mentioned lateralization issues could be evidenced by the results concerning the occlusal load distribution (Table 5, Table 6 and Table 7). In the entire study group as well as in the group of females and group of males, a greater concentration of occlusal forces was observed on the left side of clenched dental arches (Table 5, Table 6 and Table 7). No statistically significant differences were observed with regard to gender (Table 8). The asymmetry of occlusal force distribution may indicate that the source of dysfunction might be either a one-sided chewing pattern or one of disorders closely related to the lateralization of the so-called descending or ascending cranio-mandibular, homo- or heterolateral dysfunction [44]. In such cases, T-scan III could be used as an occlusal biofeedback employed to facilitate engrams involved in bite dynamics. In this aspect, further research should be conducted on the possibilities of preserving the occlusal force center, force development, and movement of the center of force as well as the center of gravity [45].

Proper dental occlusion facilitates chewing performance and adequate stimulation of the nervous system. Occlusion enables mastication, and mastication, in turn, determines sensory amplification. In the latest research, a direct link between mastication and cognitive function is strongly highlighted. This dependence is defined as the brain-stomatognathic axis [46]. As a physiological process, mastication reflects a complex movement of a neuronal network, which affects some regions of the brain, including the prefrontal cortex [47,48,49]. Typically, increased cortical blood flow is observed within the somatosensory and motor region, insular cortex, thalamus, corpus striatum, cerebellum, and hippocampus [47]. Studies on animal models indicated that mastication can alter neuronal metabolism. In rats, molar dysfunction results in progressive loss of memory and learning ability, which suggests deficits in cognitive decline [50,51]. After multiple tooth loss, the subsequent rewiring, remapping, and rebuilding of the sensorimotor details in accordance with the originally determined neuromuscular pathways are impaired [52,53]. Occlusal disharmony leads to hippocampal morphological and functional disturbances [46]. The activity of the hippocampus is conditioned by the noradrenergic, serotonergic, and dopaminergic innervation. Therefore, changes caused by occlusal disharmony may affect the function of the hippocampus. Some authors indicated that an increase in vertical occlusal dimension with acrylic by 0.1 mm results in changes within the central nervous system, which can accelerate involutional changes associated with the hippocampus-related cognitive function [46]. According to recent considerations, food properties can also influence the afferent input signal in the central nervous system. A type of food determines chewing forces, vertical and lateral movements of the mandible, rate of masticatory cycles, and frequency of mastication [54]. Organoleptic characteristics of the food are monitored by the brainstem. Food properties significantly affect the subjective perception and the so-called mouthfeel. A significant role is attributed to mechanical properties (elasticity, hardness, cohesiveness, breaking resistance), wetness (moisture, absorption, release of liquid), structure (granularity, softness, viscosity, graininess), chewing sensation (chewability, adhesion, stickiness, slipperiness, roughness, heaviness), and chewing experience (uniformity of the bite, uniformity of chewing, texture, overall uniformity, dryness) [54]. The enormity of the related stimuli determines somatosensory amplification. Furthermore, the latest research revealed that the overall oral tactile acuity is increased in patients with painful TMD, which could suggest elevated vulnerability to occlusal alterations [55]. Bucci et al. demonstrated that increased oral tactile acuity is treated as a risk factor connected with occlusal dysaesthesia under occlusal hypervigilance conditions which, in turn, are insufficient to trigger TMDs alone. These authors stressed that self-assessment by the patient is more important than the degree of somatosensation—“occlusal scanning” [55].

Occlusally determined mastication might be a preventive factor against neurodegenerative disorders [56]. It has been evidenced that tooth loss increases the risk of senile dementia or Alzheimer disorder [46]. In an animal model of Alzheimer’s disease (rats), prolonged soft food diet resulted in decreased neuronal proliferation determined by the hippocampus as well as impaired memory and learning capacity [46].

Bearing in mind the aforementioned dependencies and the fact that occlusion is currently perceived as a broader neurophysiological concept, prosthetic rehabilitation—as a global impact on the functioning of the human body—should involve anterior guidance, stable bilateral tooth contacts in maximum intercuspation and centric relation, proper distribution of contacts in maximum intercuspation, adequate axially directed occlusal forces, freedom of contact movements oscillating from maximum intercuspation, and lack of damaging intermaxillary contacts during lateral and protrusive movements [2]. In the era of new clinical challenges, developing our knowledge about the neurophysiological model of mastication, constant evaluation of bite dynamics, as well as consideration of the masticatory organ in the context of biotensegrity seem to be issues of the utmost importance.

### Strengths and Limitations of the Study

The presented research and other clinical studies based on DC/TMD will enable the selection of a homogeneous group of patients with respect to strictly defined research criteria. This, in turn, promotes a better understanding between scientists working on similar topics.

This is probably the first study on biotensegrity in the context of soft tissue mobilization in patients with temporomandibular joint disorders—myofascial pain with referral as well as the first research identifying the role and effect of soft tissue mobilization and tensegrity on occlusal parameters.

The applied clinical procedure has confirmed the possibility of using instrumental analysis in connection with a new independent research axis with respect to the DC/TMD. It should be highlighted that Axis III of the DC/TMD was designed for relevant biomarkers (quantitative sensory measures and genomic or molecular profiles), and axis IV was developed for the classification of the patients into clinically meaningful categories [10].

The main advantage of the T-scan III system is the fact that it is a widely used, reproducible, and non-invasive method. The precision of each single registration includes the coverage factor 1.96 (measurement error). The overall accuracy (repeatability, reproducibility) of the subsequent measurements involves factor 2.77. In the case of the maximum total force measured, the error amounts to 1%, reliability is 2.8%, and accuracy reaches 2% [57,58].

Although in the presented study the tested occlusal parameters oscillated within reference values, there is a clinical need to perform another research in the future aimed at analyzing the individual biting dynamics for each case. It may demonstrate that the diagnosed myofascial pain with referral is closely correlated with the presence of even premature occlusal contact.

T-scan III does not consider biodynamics of mastication which—as shown above—is an important factor in the brain-stomatognathic system axis. Apart from occlusal parameters, patients should be tested for their chewing efficiency, mastication time, occlusal forces involved in chewing, and the time of contact between both dental arches. Perhaps a possibility of 24 h evaluation of the bite dynamics would enable us to assess the degree of somatosensation and somatosensory amplification.

Another limitation is the fact that there is no objective tool that would allow for direct monitoring of the changes in biotensegrity.

## 5. Conclusions

The occlusion time and both right and left disclusion times cannot be considered as cofactors of the existing temporomandibular disorders—myofascial pain with referral.

Soft tissue mobilization appears to be effective in reduction of the occlusion and disclusion times. These findings suggest caution in occlusal equilibration with respect to TMDs management. The hypothesis that soft tissue mobilization could affect the occlusal load distribution has not been confirmed. Due to the small sample size, such effect cannot be excluded. Further research in this area are needed.

## Figures and Tables

**Table 1:** Occlusion time (s), right disclusion time (s), and left disclusion time (s) over three weeks in the entire study group (*n* = 50). Mean values, standard deviation (±SD), median (Me) and *p*-value are given. Results of the Friedman test and post hoc tests are presented.

Variables	Before 1st Treatment			After 1st Treatment			Before 2nd Treatment			After 2nd Treatment			Before 3rd Treatment			After 3rd Treatment		
	Mean	±SD	Me	Mean	±SD	Me	Mean	±SD	Me	Mean	±SD	Me	Mean	±SD	Me	Mean	±SD	Me
TO	0.191	0.073	0.175	0.151	0.048	0.140	0.145	0.046	0.140	0.134	0.044	0.130	0.154	0.116	0.130	0.119	0.036	0.110
TDR	0.209	0.061	0.210	0.181	0.052	0.185	0.173	0.056	0.170	0.164	0.051	0.160	0.167	0.043	0.160	0.159	0.045	0.155
TDL	0.214	0.071	0.210	0.185	0.055	0.185	0.176	0.044	0.170	0.179	0.050	0.170	0.170	0.043	0.170	0.165	0.039	0.165
							Post hoc tests/Pairwise comparisons with the Bonferroni correction											
	Friedman test						Kendall’s coefficient of concordance		Before 1st and after 1st treatment		Before 1st and after 2nd treatment		Before 1st and after 3rd treatment		Before 2nd and after 2nd treatment		Before 3rd and after 3rd treatment	
	Friedman statistic (F_r_)			df	*p*-value		W		*p*-value		*p*-value		*p*-value		*p*-value		*p*-value	
TO	66.545455			5	0.00000 *		0.277270		0.000812 **		0.000001 **		0.000000 **		1.000000		0.057532	
TDR	39.462560			5	0.00000 *		0.161072		0.195002		0.000026 **		0.000004 **		1.000000		1.000000	
TDL	29.431818			5	0.00002 *		0.120130		0.375596		0.063200		0.000162 **		1.000000		1.000000	

* *p* < 0.05 statistical significance; ** *p* < 0.05 statistical significance adjusted to the Bonferroni correction; *Functional Occlusal Parameters:* Occlusion time (OT)—the time from the moment of the first contact of the upper and lower teeth to the maximum occlusion of the dental arches (the standard in the case of natural dentition is <0.2 s); Right disclusion time (TDR)—the time from the moment of maximum clenching of both dental arches to their complete disengagement with lateral movement to the right (the standard in the case of natural dentition is <0.4 s); Left disclusion time (TDL)—the time from the moment of maximum intercuspation of both dental arches to their complete disassembly with lateral movement towards the left (the norm in the case of natural dentition is <0.4 s).

**Table 2:** Occlusion time (s), right disclusion time (s) and left disclusion time (s) over 3 weeks in women (*n* = 37). Mean values, standard deviation (±SD), median (Me), and *p*-value are given. Results of the Friedman test and post hoc tests are presented.

Variables	Before 1st Treatment			After 1st Treatment			Before 2nd Treatment			After 2nd Treatment			Before 3rd Treatment			After 3rd Treatment		
	Mean	±SD	Me	Mean	±SD	Me	Mean	±SD	Me	Mean	±SD	Me	Mean	±SD	Me	Mean	±SD	Me
TO	0.182	0.057	0.170	0.152	0.048	0.140	0.141	0.041	0.140	0.134	0.041	0.130	0.155	0.132	0.130	0.115	0.034	0.100
TDR	0.200	0.051	0.200	0.186	0.047	0.190	0.165	0.046	0.160	0.162	0.054	0.160	0.160	0.036	0.150	0.152	0.039	0.140
TDL	0.206	0.058	0.210	0.187	0.053	0.190	0.173	0.043	0.170	0.177	0.047	0.170	0.165	0.041	0.170	0.161	0.037	0.160
							Post hoc tests/Pairwise comparisons with the Bonferroni correction											
	Friedman test						Kendall’s coefficient of concordance		Before 1st and after 1st treatment		Before 1st and after 2nd treatment		Before 1st and after 3rd treatment		Before 2nd and after 2nd treatment		Before 3rd and after 3rd treatment	
	Friedman statistic (F_r_)			df	*p*-value		W		*p*-value		*p*-value		*p*-value		*p*-value		*p*-value	
TO	50.645592			5	0.000000 *		0.28940		0.015017 **		0.000116 **		0.000000 **		1.000000		0.090170	
TDR	34.822754			5	0.000002 *		0.19346		1.000000		0.002356 **		0.000047 **		1.000000		1.000000	
TDL	23.351464			5	0.000289 *		0.12973		0.474535		1.000000		0.540475		1.000000		1.000000	

* *p* < 0.05 statistical significance; ** *p* < 0.05 statistical significance adjusted to the Bonferroni correction; Functional occlusal parameters: Occlusion time (OT)—the time from the moment of the first contact of the upper and lower teeth to the maximum occlusion of the dental arches (the standard in the case of natural dentition is <0.2 s); Right disclusion time (TDR) —the time from the moment of maximum clenching of both dental arches to their complete disengagement with lateral movement to the right (the standard in the case of natural dentition is <0.4 s); Left disclusion time (TDL)—the time from the moment of maximum intercuspation of both dental arches to their complete disassembly with lateral movement towards the left (the norm in the case of natural dentition is <0.4 s).

**Table 3:** Occlusion time (s), right disclusion time (s) and left disclusion time (s) over three weeks in men (*n* = 13). Mean values, standard deviation (± SD), median (Me), and *p*-value are given. Results of the Friedman test and post hoc tests are presented.

Variables	Before 1st Treatment			After 1st Treatment			Before 2nd Treatment			After 2nd Treatment			Before 3rd Treatment			After 3rd Treatment		
	Mean	±SD	Me	Mean	±SD	Me	Mean	±SD	Me	Mean	±SD	Me	Mean	±SD	Me	Mean	±SD	Me
TO	0.216	0.105	0.180	0.145	0.049	0.140	0.159	0.059	0.140	0.132	0.051	0.130	0.149	0.054	0.140	0.129	0.042	0.130
TDR	0.236	0.078	0.250	0.165	0.063	0.160	0.195	0.076	0.180	0.169	0.043	0.150	0.185	0.055	0.160	0.180	0.057	0.180
TDL	0.239	0.098	0.210	0.182	0.062	0.170	0.185	0.048	0.180	0.186	0.060	0.180	0.182	0.048	0.170	0.177	0.045	0.180
									Post hoc tests/Pairwise comparisons with the Bonferroni correction									
	Friedman test						Kendall’s coefficient of concordance		Before 1st and after 1st treatment		Before 1st and after 2nd treatment		Before 1st and after 3rd treatment		Before 2nd and after 2nd treatment		Before 3rd and after 3rd treatment	
	Friedman statistic (F_r_)			df	*p*-value		W		*p*-value		*p*-value		*p*-value		*p*-value		*p*-value	
TO	16.942446			5	0.004610 *		0.260653		0.275130		0.035484 **		0.004478 **		1.000000		1.000000	
TDR	10.902935			5	0.053339		0.167737		0.238597		0.042175 *		0.415618		1.000000		1.000000	
TDL	8.579677			5	0.127050		0.131995		0.474535		1.000000		0.540475		1.000000		1.000000	

* *p* < 0.05 statistical significance; ** *p* < 0.05 statistical significance adjusted to the Bonferroni correction.

**Table 4:** Comparison of occlusion time (s), right disclusion time (s), and left disclusion time (s) with respect to gender over three weeks. Results of the Mann–Whitney U Test are presented (*p*-value).

	Mann–Whitney U Test Comparing Average Values of TO, TDR, TDL between Females and Males before and after Each Treatment					
Variable	Before 1st Treatment	After 1st Treatment	Before 2nd Treatment	After 2nd Treatment	Before 3rd Treatment	After 3rd Treatment
	*p*-value	*p*-value	*p*-value	*p*-value	*p*-value	*p*-value
TO	0.5495	0.6728	0.4493	0.6475	0.4823	0.2414
TDR	0.1345	0.1624	0.2922	0.7902	0.2036	0.0569
TDL	0.4783	0.8767	0.4229	0.8763	0.3671	0.3505

Occlusion time (OT)—the time from the moment of the first contact of the upper and lower teeth to the maximum occlusion of the dental arches (the standard in the case of natural dentition is <0.2 s); Right disclusion time (TDR)—the time from the moment of maximum clenching of both dental arches to their complete disengagement with lateral movement to the right (the standard in the case of natural dentition is <0.4 s); Left disclusion time (TDL)—the time from the moment of maximum intercuspation of both dental arches to their complete disassembly with lateral movement towards the left (the norm in the case of natural dentition is <0.4 s).

**Table 5:** Occlusal load (%) on the right and left side of dental arches in maximal intercuspation over three weeks in the entire study group (*n* = 50). Mean values, standard deviation (± SD), and median (Me) are given. Results of one-way repeated measures ANOVA and post hoc Tukey’s comparison test are presented.

Variables	Before 1st Treatment			After 1st Treatment			Before 2nd Treatment			After 2nd Treatment			Before 3rd Treatment			After 3rd Treatment		
	Mean	±SD	Me	Mean	±SD	Me	Mean	±SD	Me	Mean	±SD	Me	Mean	±SD	Me	Mean	±SD	Me
Occlusal load on the right side	47.2	8.3	46.2	48.2	7.5	48.5	46.4	9.6	45.3	47.9	8.8	48.0	47.8	8.3	47.6	47.5	6.9	48.5
Occlusal load on the left side	52.8	8.2	53.9	51.8	7.5	51.6	53.6	9.5	54.7	52.1	8.8	52.1	52.2	8.3	52.5	52.5	6.9	51.6
							Post hoc Tukey’s comparison test											
	Repeated measures of one-way ANOVA								Before 1st and after 1st treatment		Before 1st and after 2nd treatment		Before 1st and after 3rd treatment		Before 2nd and after 2nd treatment		Before 3rd and after 3rd treatment	
	Groups		Sum of square	df	Mean square	F	*p*-value	R squared	*p*-value		*p*-value		*p*-value		*p*-value		*p*-value	
Occlusal load on the right side	Between columns		102.5	5	20.51	0.574	0.6720	0.01158	0.9786		0.9930		0.9999		0.7282		0.9998	
	Between rows		11,439	49	233.4	6.534	<0.0001 *											
	Residual (random)		8754	245	35.73													
	Total		20,295	299														
Occlusal load on the left side	Between columns		108.4	5	21.68	0.617	0.6451	0.01243	0.9491		0.9753		0.9980		0.7331		0.9998	
	Between rows		11,446	49	233.6	6.645	<0.0001 *											
	Residual (random)		8613	245	35.16													
	Total		20,168	299														

* *p* < 0.05 statistical significance.

**Table 6:** Occlusal load (%) on the right and left side of dental arches in maximal intercuspation over three weeks in females (*n* = 37). Mean values, standard deviation (± SD) and median (Me) are given. Results of repeated measures of ANOVA and post hoc tests are presented.

Variables	Before 1st Treatment			After 1st Treatment			Before 2nd Treatment			After 2nd Treatment			Before 3rd Treatment			After 3rd Treatment		
	Mean	±SD	Me	Mean	±SD	Me	Mean	±SD	Me	Mean	±SD	Me	Mean	±SD	Me	Mean	±SD	Me
Occlusal load on the right side	47.7	8.3	46.8	47.8	7.4	47.7	46.9	9.5	47.6	48.5	9.6	47.9	48.3	8.9	48.0	47.3	7.6	47.0
Occlusal load on the left side	52.3	8.3	53.2	52.2	7.4	52.3	53.1	9.5	52.4	51.5	9.6	52.1	51.7	8.9	52.0	52.7	7.6	53.0
							Post hoc Tukey’s comparison test											
	Repeated measures of one-way ANOVA								Before 1st and after 1st treatment		Before 1st and after 2nd treatment		Before 1st and after 3rd treatment		Before 2nd and after 2nd treatment		Before 3rd and after 3rd treatment	
	Groups		Sum of square	df	Mean square	F	*p*-value	R squared	*p*-value		*p*-value		*p*-value		*p*-value		*p*-value	
Occlusal load on the right side	Between columns		65.31	5	13.06	0.3891	0.8195	0.01069	>0.9999		0.9932		0.9997		0.7945		0.9734	
	Between rows		9979	36	277.2	8.258	<0.0001 *											
	Residual (random)		6042	180	33.57													
	Total		16,086	221														
Occlusal load on the left side	Between columns		64.44	5	12.89	0.3842	0.8232	0.01056	>0.9999		0.9933		0.9991		0.7997		0.9734	
	Between rows		9964	36	276.8	8.250	<0.0001 *											
	Residual (random)		6039	180	33.55													
	Total		16,067	221														

* *p* < 0.05 statistical significance.

**Table 7:** Occlusal load (%) on the right and left side of dental arches in maximal intercuspation over three weeks in males (*n* = 13). Mean values, standard deviation (± SD), and median (Me) are given. Results of the Friedman test and post hoc tests are presented.

Variables	Before 1st Treatment			After 1st Treatment			Before 2nd Treatment			After 2nd Treatment			Before 3rd Treatment			After 3rd Treatment		
	Mean	±SD	Me	Mean	±SD	Me	Mean	±SD	Me	Mean	±SD	Me	Mean	±SD	Me	Mean	±SD	Me
Occlusal load on the right side	45.7	8.6	45.8	49.2	7.9	49.1	44.9	10.0	45.0	46.3	6.1	48.0	46.3	6.2	47.0	48.0	4.6	48.9
Occlusal load on the left side	54.3	7.9	54.2	50.8	7.9	50.9	55.1	10.0	55.0	53.7	6.1	52.0	53.7	6.2	53.0	52.0	4.6	51.1
							Post hoc tests/Pairwise comparisons with the Bonferroni correction											
	Friedman test						Kendall’s coefficient of concordance		Before 1st and after 1st treatment		Before 1st and after 2nd treatment		Before 1st and after 3rd treatment		Before 2nd and after 2nd treatment		Before 3rd and after 3rd treatment	
	Friedman statistic (F_r_)			df	*p*-value		W		*p*-value		*p*-value		*p*-value		*p*-value		*p*-value	
Occlusal load on the right side	7.898230			5	0.161934		0.121511		1.000000		1.000000		1.000000		1.000000		1.000000	
Occlusal load on the left side	9.446903			5	0.092512		0.145337		0.887581		1.000000		0.540475		1.000000		1.000000	

**Table 8:** Comparison of occlusal load (%) on the right and left side of dental arches in maximal intercuspation over three weeks with respect to gender. Results of the Mann-Whitney U test are presented (*p*-value).

Variable	Mann-Whitney U Test Comparing the Average Value of Occlusal Loads between Females and Males before and after Each Treatment					
	Before 1st Treatment	After 1st Treatment	Before 2nd Treatment	After 2nd Treatment	Before 3rd Treatment	After 3rd Treatment
	*p*-value	*p*-value	*p*-value	*p*-value	*p*-value	*p*-value
Occlusal load on the left side	0.2640	0.4790	0.4999	0.5430	0.4006	0.6662
Occlusal load on the right side	0.3945	0.4790	0.4999	0.5430	0.4006	0.6662

## Data Availability

The article contains complete data used to support the findings of this study.

## References

[1] Abdi A.H., Sagl B., Srungarapu V.P., Stavness I., Prisman E., Abolmaesumi P., Fels S. (2020). Characterizing Motor Control of Mastication With Soft Actor-Critic. Front. Hum. Neurosci..

[2] Peck C. (2016). Biomechanics of occlusion–implications for oral rehabilitation. J. Oral Rehabil..

[3] Seok H., Kim S.-G. (2018). Correction of malocclusion by botulinum neurotoxin injection into masticatory muscles. Toxins.

[4] Svensson P., Graven-Nielsen T. (2001). Craniofacial muscle pain: Review of mechanisms and clinical manifestations. J. Orofac. Pain.

[5] Michelotti A., Rongo R., D’Antò V., Bucci R. (2020). Occlusion, orthodontics, and temporomandibular disorders: Cutting edge of the current evidence. J. World Fed. Orthod..

[6] Al-Ani Z. (2020). Occlusion and Temporomandibular Disorders: A Long-Standing Controversy in Dentistry. Prim. Dent. J..

[7] Palla S. (2005). The interface of occlusion as a reflection of conflicts within prosthodontics. Int. J. Prosthodont..

[8] Michelotti A., Alstergren P., Goulet J., Lobbezoo F., Ohrbach R., Peck C., Schiffman E., List T. (2016). Next steps in development of the diagnostic criteria for temporomandibular disorders (DC/TMD): Recommendations from the International RDC/TMD Consortium Network workshop. J. Oral Rehabil..

[9] Ohrbach R., Gonzalez Y., List T., Michelotti A., Schiffman E. (2014). Diagnostic Criteria for Temporomandibular Disorders (DC/TMD) Clinical Examination Protocol. www.rdc-tmdinternational.org.

[10] Schiffman E., Ohrbach R., Truelove E., Look J., Anderson G., Goulet J.-P., List T., Svensson P. (2014). Diagnostic criteria for temporomandibular disorders (DC/TMD) for clinical and research applications: Recommendations of the International RDC/TMD Consortium Network and Orofacial Pain Special Interest Group. J. Oral Facial Pain Headache.

[11] Schiffman E., Ohrbach R. (2016). Executive summary of the Diagnostic Criteria for Temporomandibular Disorders for clinical and research applications. J. Am. Dent. Assoc..

[12] Dalewski B., Kamińska A., Szydłowski M., Kozak M., Sobolewska E. (2019). Comparison of early effectiveness of three different intervention methods in patients with chronic orofacial pain: A randomized, controlled clinical trial. Pain Res. Manag..

[13] Li D.T.S., Leung Y.Y. (2021). Temporomandibular Disorders: Current Concepts and Controversies in Diagnosis and Management. Diagnostics.

[14] Lindfors E., Arima T., Baad-Hansen L., Bakke M., De Laat A., Giannakopoulos N.N., Glaros A., Guimarães A.S., Johansson A., Le Bell Y. (2019). Jaw Exercises in the Treatment of Temporomandibular Disorders--An International Modified Delphi Study. J. Oral Facial Pain Headache.

[15] Armijo-Olivo S., Pitance L., Singh V., Neto F., Thie N., Michelotti A. (2016). Effectiveness of manual therapy and therapeutic exercise for temporomandibular disorders: Systematic review and meta-analysis. Phys. Ther..

[16] Abouelhuda A.M. (2018). Non-invasive different modalities of treatment for temporomandibular disorders: Review of literature. J. Korean Assoc. Oral Maxillofac. Surg..

[17] Nitecka-Buchta A., Nowak-Wachol A., Wachol K., Walczyńska-Dragon K., Olczyk P., Batoryna O., Kempa W., Baron S. (2019). Myorelaxant Effect of Transdermal Cannabidiol Application in Patients with TMD: A Randomized, Double-Blind Trial. J. Clin. Med..

[18] Espí-López G.V., Arnal-Gómez A., Cuerda del Pino A., Benavent-Corai J., Serra-Añó P., Inglés M. (2020). Effect of manual therapy and splint therapy in people with temporomandibular disorders: A preliminary study. J. Clin. Med..

[19] Scarr G., Harrison H. (2017). Examining the temporo-mandibular joint from a biotensegrity perspective: A change in thinking. J. Appl. Biomed..

[20] Zarone F., Apicella A., Nicolais L., Aversa R., Sorrentino R. (2003). Mandibular flexure and stress build-up in mandibular full-arch fixed prostheses supported by osseointegrated implants. Clin. Oral Implant. Res..

[21] Daegling D.J. (2012). The human mandible and the origins of speech. J. Anthropol..

[22] Consolaro A., Cardoso M.d.A. (2018). Mandibular anterior crowding: Normal or pathological?. Dent. Press J. Orthod..

[23] Scarr G., Harrison H. (2016). Resolving the problems and controversies surrounding temporo-mandibular mechanics. J. Appl. Biomed..

[24] Scarr G. (2020). Biotensegrity: What is the big deal?. J. Bodyw. Mov. Ther..

[25] Kuć J., Szarejko K.D., Gołębiewska M. (2020). Evaluation of Soft Tissue Mobilization in Patients with Temporomandibular Disorder-Myofascial Pain with Referral. Int. J. Environ. Res. Public Health.

[26] Kuć J., Szarejko K.D., Sierpinska T. (2019). Evaluation of Orofacial and General Pain Location in Patients With Temporomandibular Joint Disorder-Myofascial Pain With Referral. Front. Neurol..

[27] Kuć J., Szarejko K.D., Gołȩbiewska M. (2021). Smiling, Yawning, Jaw Functional Limitations and Oral Behaviors with Respect to General Health Status in Patients With Temporomandibular Disorder—Myofascial Pain With Referral. Front. Neurol..

[28] Kerstein R.B. (2010). Reducing chronic masseter and temporalis muscular hyperactivity with computer-guided occlusal adjustments. Compend Contin Educ. Dent..

[29] Kerstein R.B. (2004). Combining technologies: A computerized occlusal analysis system synchronized with a computerized electromyography system. Cranio^®^.

[30] Kerstein R.B., Radke J. (2006). The effect of disclusion time reduction on maximal clench muscle activity levels. Cranio^®^.

[31] Simons D.G., Travell J.G., Simons L.S. (1999). Travell & Simons’ Myofascial Pain and Dysfunction: Upper Half of Body.

[32] Aggarwal A., Gadekar J., Kakodkar P. (2020). Role of myofascial release technique on mobility and function in temporomandibular joint disorder patients with neck pain. J. Dent. Res. Rev..

[33] Lerman M.D. (2011). The muscle engram: The reflex that limits conventional occlusal treatment. Cranio^®^.

[34] Dalewski B., Chruściel-Nogalska M., Frączak B. (2015). Occlusal splint versus modified nociceptive trigeminal inhibition splint in bruxism therapy: A randomized, controlled trial using surface electromyography. Aust. Dent. J..

[35] Florjanski W., Malysa A., Orzeszek S., Smardz J., Olchowy A., Paradowska-Stolarz A., Wieckiewicz M. (2019). Evaluation of biofeedback usefulness in masticatory muscle activity management—A systematic review. J. Clin. Med..

[36] Pfeiffer K., El Khassawna T., Malhan D., Langer C., Sommer B., Mekhemar M., Howaldt H.-P., Attia S. (2021). Is Biofeedback through an Intra-Aural Device an Effective Method to Treat Bruxism? Case Series and Initial Experience. Int. J. Environ. Res. Public Health.

[37] Manfredini D. (2018). Occlusal equilibration for the management of temporomandibular disorders. Oral Maxillofac. Surg. Clin..

[38] Kuvatanasuchati J., Leowsrisook K. (2019). The simple treatment of chronic facial pain due to trigeminal neuralgia with dental occlusal equilibration. Interdiscip. Neurosurg..

[39] Ramachandran A., Jose R., Tunkiwala A., Varma R.B.M., Shanmugham A., Nair P.K., Kumar K.S., Sam L.M. (2019). Effect of deprogramming splint and occlusal equilibration on condylar position of TMD patients—A CBCT assessment. Cranio^®^.

[40] Koh H., Robinson P. (2004). Occlusal adjustment for treating and preventing temporomandibular joint disorders. J. Oral Rehabil..

[41] Sierpinska T., Kuc J., Golebiewska M. (2013). Morphological and functional parameters in patients with tooth wear before and after treatment. Open Dent. J..

[42] Ferrario V.F., Sforza C., Zanotti G., Tartaglia G.M. (2004). Maximal bite forces in healthy young adults as predicted by surface electromyography. J. Dent..

[43] Cuccia A., Caradonna C. (2009). The relationship between the stomatognathic system and body posture. Clinics.

[44] Dupas P.-H. (2005). Nouvelle Approche du Dysfonctionnement Cranio-Mandibulaire: Du Diagnostic à la Gouttière.

[45] Westersund C.D., Scholten J., Turner R.J. (2017). Relationship between craniocervical orientation and center of force of occlusion in adults. Cranio^®^.

[46] Iwayama J.J. (2020). Masticatory Deficiency and Deficit of Cognitive Function, Attention, Learning and Memory. Ec. Dent. Sci..

[47] Chuhuaicura P., Dias F.J., Arias A., Lezcano M.F., Fuentes R. (2019). Mastication as a protective factor of the cognitive decline in adults: A qualitative systematic review. Int. Dent. J..

[48] Lin C.-s. (2018). Revisiting the link between cognitive decline and masticatory dysfunction. BMC Geriatr..

[49] Yokoyama T., Sato M., Natsui S., Kuboyama N., Suzuki K., Inaba H., Shibuya K. (2017). Effect of gum chewing frequency on oxygenation of the prefrontal cortex. Percept. Mot. Ski..

[50] Yamamoto T., Hirayama A. (2001). Effects of soft-diet feeding on synaptic density in the hippocampus and parietal cortex of senescence-accelerated mice. Brain Res..

[51] Terasawa H., Hirai T., Ninomiya T., Ikeda Y., Ishijima T., Yajima T., Hamaue N., Nagase Y., Kang Y., Minami M. (2002). Influence of tooth-loss and concomitant masticatory alterations on cholinergic neurons in rats: Immunohistochemical and biochemical studies. Neurosci. Res..

[52] Gladding C.M., Raymond L.A. (2011). Mechanisms underlying NMDA receptor synaptic/extrasynaptic distribution and function. Mol. Cell. Neurosci..

[53] Krishnamoorthy G., Narayana A.I., Balkrishanan D. (2018). Mastication as a tool to prevent cognitive dysfunctions. Jpn. Dent. Sci. Rev..

[54] Slavicek G. (2020). The influence of occlusion on masticatory efficiency considering relevant influencing factors. Stoma Edu J..

[55] Bucci R., Koutris M., Palla S., Sepúlveda Rebaudo G.F., Lobbezoo F., Michelotti A. (2020). Occlusal tactile acuity in temporomandibular disorder pain patients: A case-control study. J. Oral Rehabil..

[56] Chen H., Iinuma M., Onozuka M., Kubo K.-Y. (2015). Chewing maintains hippocampus-dependent cognitive function. Int. J. Med. Sci..

[57] Koos B. (2017). Precision and reliability of the T-scan III system: Analyzing occlusion and the resultant timing and distribution of forces in the dental arch. Medical Imaging: Concepts, Methodologies, Tools, and Applications.

[58] Kuć J., Szarejko K.D., Aleksandrowicz K., Gołębiewska M. (2021). The role of soft tissue mobilization in reducing orofacial and general complaints in a patient with Kimmerle anomaly and temporomandibular joint disorder: A case report. Cranio^®^.

